# Association, characterisation and meta-analysis of SNPs linked to general reading ability in a German dyslexia case-control cohort

**DOI:** 10.1038/srep27901

**Published:** 2016-06-17

**Authors:** Bent Müller, Arndt Wilcke, Ivonne Czepezauer, Peter Ahnert, Johannes Boltze, Holger Kirsten, The LEGASCREEN consortium, Angela D. Friederici, Frank Emmrich, Jens Brauer, Arndt Wilcke, Nicole Neef, Johannes Boltze, Michael Skeide, Holger Kirsten, Gesa Schaadt, Bent Müller, Indra Kraft, Ivonne Czepezauer, Liane Dörr

**Affiliations:** 1 Fraunhofer Institute for Cell Therapy and Immunology, Leipzig, Germany; 2 Translational Centre for Regenerative Medicine (TRM), Leipzig, Germany; 3 Institute for Medical Informatics, Statistics and Epidemiology, University of Leipzig, Leipzig, Germany; 4 LIFE—Leipzig Research Center for Civilization Diseases, University of Leipzig, Leipzig, Germany; 5 Fraunhofer Research Institution for Marine Biotechnology, Department of Medical Cell Technology, Lübeck, Germany; 6 Institute for Medical and Marine Biotechnology, University of Lübeck, Lübeck, Germany; 7 Department of Neuropsychology, Max Planck Institute for Human Cognitive and Brain Sciences, Leipzig, Germany; 8 Department of Psychology, Humboldt-Universität zu Berlin, Berlin, Germany

## Abstract

Dyslexia is a severe disorder in the acquisition of reading and writing. Several studies investigated the role of genetics for reading, writing and spelling ability in the general population. However, many of the identified SNPs were not analysed in case-control cohorts. Here, we investigated SNPs previously linked to reading or spelling ability in the general population in a German case-control cohort. Furthermore, we characterised these SNPs for functional relevance with *in silico* methods and meta-analysed them with previous studies. A total of 16 SNPs within five genes were included. The total number of risk alleles was higher in cases than in controls. Three SNPs were nominally associated with dyslexia: rs7765678 within *DCDC2*, and rs2038137 and rs6935076 within *KIAA0319*. The relevance of rs2038137 and rs6935076 was further supported by the meta-analysis. Functional profiling included analysis of tissue-specific expression, annotations for regulatory elements and effects on gene expression levels (eQTLs). Thereby, we found molecular mechanistical implications for 13 of all 16 included SNPs. SNPs associated in our cohort showed stronger gene-specific eQTL effects than non-associated SNPs. In summary, our results validate SNPs previously linked to reading and spelling in the general population in dyslexics and provide insights into their putative molecular pathomechanisms.

Dyslexia is a neurodevelopmental disorder which affects the ability of accurate and/or fluent word recognition and spelling^1^. These problems are thought to result from deficits in phonological decoding and are independent of general intelligence. With a prevalence of ~5% in Germany, it is one of the most common learning impairments for young school children^2^. Twin studies estimated the heritability of dyslexia at 50–70%^3^. The underlying genetics is complex and appears to consist of a large number of factors with rather small effect sizes. In recent years, several candidate genes and single nucleotide polymorphisms (SNPs) associated with dyslexia were found^4^. From those genes, *DYX1C1*, *KIAA0319* and *DCDC2* are the most prominent candidate genes for dyslexia. *KIAA0319* is strongly expressed in the human cortex^5^ and knockdown in rats revealed disturbed neuronal migration in the neocrotex^6^. Similar findings were reported for *DCDC2* and *DYX1C1* where embryonic knockdown resulted in neuronal overmigration past the desired layer^6^. All three genes were reported to be linked with reduced white matter volume in the left hemisphere of dyslexics^7^, a well-known dyslexia brain phenotype. In addition to findings from dyslexia-control studies, several candidate genes were also found to be associated with reading or spelling ability in the general population^8^,^9^,^10^,^11^,^12^. These SNPs were mainly located in the DYX2 locus (*DCDC2*^8^, *KIAA0319*^13^ and *TDP*^12^ (also known as *TTRAP*)) but also variants in *ROBO1*^10^, *DYX1C1*^9^ and *NKAIN2*^11^ were identified (Table 1).

Genetic variants associating with a quantitative trait are highly relevant genetic candidate variants in a corresponding binary trait as seen in other diseases^14^. However, we identified that many of these SNPs (12 of 16 in four genes shown in Table 1) were not yet analysed in a dyslexia case-control setting, in these four genes only different SNPs were previously investigated (Supplemental Tables 1 and 2). As correlation between SNPs within the same gene can vary largely and SNPs may have independent effects due to locus heterogeneity, association results among different SNPs of the same gene may differ considerably. Furthermore, for the four of 16 SNPs case-control analyses already exist, information of additional association studies will provide valuable validation information helping to resolve partly contracting findings (Table 1).

Therefore, the primary aim of our study was to systematically investigate all these 16 SNPs in a German dyslexia case-control cohort. Previously, other reported dyslexia candidate SNPs were analysed in this cohort (or subsets of this cohort). Thereby, association was found for three of eight candidates from chromosome 18^15^, for a variant in *FOXP2*^16^, and for a large 2445 bp deletion and certain SNPs in *DCDC2*^17^ but not for a functional variant in *KIAA0319*^18^ language gene.

A secondary aim of our study addresses the lack of functional hypotheses about potential molecular risk-mechanisms of these 16 SNPs. This information is valuable for an improved understanding of a potential pathomechanisms of an observed association. Moreover, it is also important as additional evidence for the validity of an observed association. Therefore, in order to address this secondary aim, we characterise all these 16 variants using *in silico* methods on large datasets from recent available high-throughput studies.

## Results

We analysed 16 SNPs in five genes from six studies^8^,^9^,^10^,^11^,^12^,^13^ affecting reading and spelling in the general population that were genotyped in our cohort (see Table 1 for details). All SNPs were in Hardy-Weinberg-equilibrium (HWE) in controls (p-value > 0.05), the call rate for each SNP exceeded 98%, and the call rate per individual was in average 99% across all 16 SNPs.

### Associations on single marker level

On single marker level, nominal significant allelic association was observed for the minor allele of rs7765678-*DCDC2* (p-value = 0.023, odds ratio (OR) = 0.65 (0.5–0.9)) and rs6935076-*KIAA0319* (p-value = 0.023, OR = 1.25 (1.0–1.5)) (Table 2). Nominal significant genotypic association was also observed for the major homozygous state of rs2038137-*KIAA0319* (p-value = 0.036, genetic relative risk (GRR) = 1.35 (1.0–1.8)) and rs7765678-*DCDC2* (p-value = 0.035, GRR = 1.52 (1.0–2.2)). Originally reported effect size of these associations was not markedly higher than for those without association. No associations reached significance when considering Bonferroni’s correction method. For all nominally associating SNPs, the observed risk alleles were in accordance to reported associations from the general population studies. Two of the three nominally associating SNPs were previously reported in case-control settings: For rs2038137-*KIAA0319* the risk alleles were in accordance with the reported associations^19^,^20^ and for rs6935076-*KIAA0319* one study reported same allele^20^ and one study reported the opposite allele as risk allele^21^.

In literature, the haplotype rs4504469-rs2038137-rs2143340 was reported to associate with measures for reading^12^,^13^. We could not identify an association in our sample (Supplemental Table 3). No association was also observed of gene-wide haplotypes in *DCDC2, KIAA0319*, and *DYX1C1* (Supplemental Table 3).

### Meta-analysis

For four of the 16 investigated SNPs, case-control data of six different studies from a total of eight countries was available for meta-analysis^19^,^20^,^21^,^22^,^23^,^24^ (Table 1). These four SNPs included nominally associated variants rs2038137-*KIAA0319* and rs693507-*KIAA0319* but not rs7765678-*DCDC2.*

**Figure 1:**
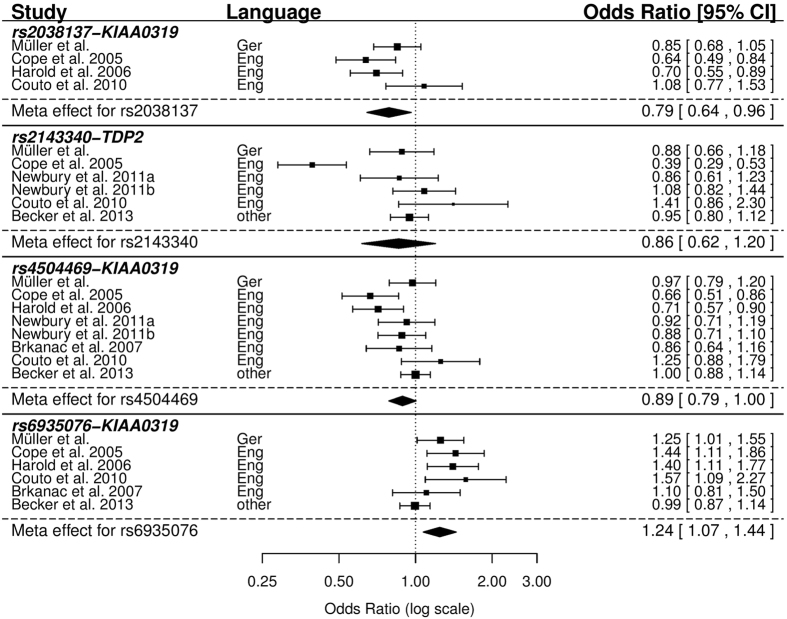
Forest plot representing the individual results of seven studies and the meta-effects for each SNP. Note, that Brkanac *et al*.^22^ and Couto *et al*.^21^ performed TDT-studies and ORs were computed from transmitted allele counts.

In line with our findings from our cohort alone, we found a significant meta-effect for SNPs rs2038137 and for rs6935076 (OR = 0.79 (0.64–0.96); p-value = 0.019 and OR = 1.24 (1.07–1.44); p-value = 0.004, respectively) across all studies (Fig. 1). For rs4504469-*KIAA0319*, only a trend-level significant meta-effect was found (p = 0.059). However, when stratifying the meta-analysis for languages, a significant meta-effect was found for this variant within English-speaking cohorts (OR = 0.84 (0.73–0.96); p-value = 0.009, Supplemental Fig. 1). No meta-effect was found for rs2143340-*TDP2*.

### Polygenic analyses

**Figure 2:**
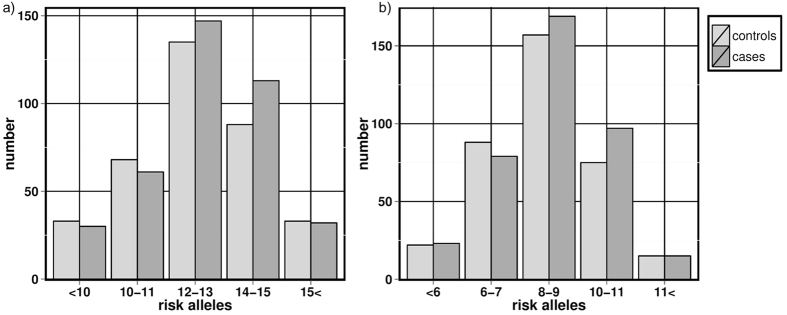
Distribution of risk alleles among dyslexia-cases and controls. (**a**) Cases and controls with the total sum of all reported risk alleles from all included 11 independent SNPs. (**b**) Cases and controls with the total sum of all reported risk alleles but excluding all three SNPs associating at single marker level in our cohort.

Next, we aimed to investigate effects that may not have been detectable in our single marker analysis. Therefore, we jointly analysed association of all 16 general-reading SNPs by creating a score summing individual-wise all risk alleles. In order to avoid bias due to linkage disequilibrium among these SNPs, we thereby considered the subset of 11 unlinked SNPs (see Material and Methods). We found that for nine of these 11 SNPs effect direction was concordant with the literature. Thereby, the reported risk alleles were increased in our cases. This was more than expected by chance (p-value = 0.033). Accordingly, an increased number of risk variants was observable within cases compared to controls (Fig. 2a). An increased number of reported risk variants in cases was also observable when including only the SNPs not associating on single marker level (Fig. 2b). However, a formal quantitative association analysis of the corresponding risk score did not meet significance (p-value > 0.2). Findings were similar when jointly considering these eleven SNPs and 14 additional genetic dyslexia candidate variants analysed in previous association studies of our cohort (Supplemental Fig. 2).

### Functional analyses

**Figure 3:**
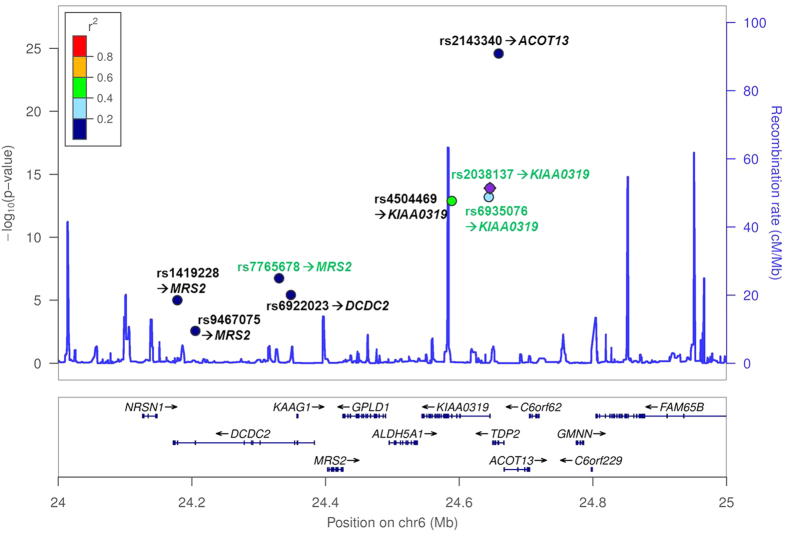
Local association plot of analysed DYX2-SNPs with reported eQTL-effect. Depicted are the analysed SNPs of DYX2 whereby SNPs with nominal significant associations with dyslexia in our sample are coloured in green. Next to each SNP following the arrow we report the gene whose expression level was strongest associated with the SNP. P-values represent the association of the SNP with gene expression levels. Details can be found in Supplemental Table 4. The data points are coloured according to the linkage to the SNP with the strongest effect in the association and the meta-analysis (rs2038137).

SNPs were evaluated for expression quantitative trait loci (eQTL)-effects by screening published eQTL-analyses. In total, twelve of all 16 SNPs showed a direct effect on expression levels of neighbouring genes (*cis*-eQTLs, Table 3). For 14 SNPs, proxy SNPs (R^2^ ≥ 0.5) were observed to be associated with the expression of a total of 16 genes in *cis* as well as in *trans (*see Table 3 and Supplemental Table 4). For *DCDC2, KIAA0319*, and *DYX1C1*, SNPs located within these genes also affected the expression of *DCDC2, KIAA0319*, and *DYX1C1*, respectively. This included nominally associated variants rs2038137 and rs693507 in *KIAA0319* that actually affected gene expression of *KIAA0319*. However, nominally associated SNP rs7765678-*DCDC2* was most strongly linked with the expression of *MRS2.* An overview of the eQTL effects of the SNPs from DYX2 (*DCDC2*, *KIAA0319* and *TDP2*) is provided in Fig. 3. Consistently, this figure demonstrates that SNPs associated in our cohort showed stronger gene-specific eQTL effects than non-associated SNPs.

To obtain complementary functional information for eQTLs, we analysed information regarding gene expression available in database ‘RegulomeDB’^25^ (Table 3). For SNP rs2038137-*KIAA0319*, we found the highest evidence of altered transcription factor binding. Ten of 16 SNPs revealed minor evidence to affect binding of transcription related factors, for five SNPs no data was available. All investigated genes, except *TDP2*, were expressed in brain according to the ‘Human Protein Atlas’^26^ (Supplemental Table 5).

## Discussion

In this study, we investigated 16 candidate SNPs representing 11 independent genetic effects reported to alter reading or spelling ability in the general population. We analysed the relevance of these variants in a German-speaking dyslexia specific case-control cohort and meta-analysed our findings. Additionally, we characterised these SNPs for molecular evidence in order to get insights into potential molecular risk-mechanisms underlying these genetic variations.

Associations in case-control studies can provide evidence for additional, epidemiological features of risk variants described for the general population. Compared to studies of the general population, case-control studies are enriched for severe cases. Therefore, the relevance of such risk variants for rather extreme, but clinically highly relevant phenotypes is of interest. This investigation may prioritise variants that not only modify the normal range of reading/spelling but are also critical for the extreme ends.

To our knowledge, from all 16 investigated SNPs only four were yet analysed in case-control settings (rs4504469-*KIAA0319*, rs2038137-*KIAA01319*, rs6935076-*KIAA0319*, rs2143340-*TDP2*, Table 1). From those four SNPs, we could nominally replicate rs2038137-*KIAA0319* and rs6935076-*KIAA0319* in a genuine German dyslexia population and confirm this association in our meta-analysis (Fig. 1). Both SNPs were previously described for English speaking dyslexics and the latter also for a mixed central European population^19^,^20^,^21^,^23^,^24^,^27^. We did not find evidence that these associations are explained by rs9451046, a previously reported functional variant in *KIAA0319* as linkage disequilibrium of our associated SNPs and this variant is rather weak (R^2^ = 0.22). In line with this, a direct association analysis of rs9451046 investigated previously in our cohort did not find a significant associations^17^,^18^. Contrarily, associating SNP rs2038137-*KIAA0319* is in perfect LD (R^2^ = 1) with rs2179515 reported in the NeuroDys study^23^, therefore both associations refer to the same genetic phenomenon (Supplemental Table 1).

The third investigated variant in *KIAA0319*, rs4504469, was not associated in our cohort and reached only trend-level significance in the meta-analysis across all studies. Interestingly, when stratifying the meta-analysis for language, we found significant association of this variant among English speaking cohorts (Supplemental Fig. 1a). Therefore, we speculate that this SNP is more relevant in the development of dyslexia in English speaking humans. Indeed, regarding the language, the German language is a relatively transparent language with a clear grapheme-phoneme correspondence compared to English^28^. These differences may lead to the involvement of different brain regions, different neuronal networks and, consequently, different contributing genetic factors for speech processing^29^. For SNP rs4504469-*KIAA0319*, results from our meta-analysis helped to identify, whether non-replication in our cohort is more likely due to language differences or due to the described differences between case-control studies and studies in the general population. For the other SNPs not associated in our cohort, we do not have external data to perform a similar meta-analysis and therefore cannot resolve this ambiguity.

Our *in silico* characterisation on the molecular, functional level using ‘RegulomeDB’ further supported especially associated SNP rs2038137-*KIAA0319.* For this SNP strongest evidence for functionality was found in this database (Table 3): This SNP affected binding of the transcription factor *RFX3*^30^, required for corpus callosum development^31^. In humans, corpus callosum deficits could lead to impaired motor coordination and balance problems, attributes known for dyslexics^32^. *RFX3* was also associated with relative hand skill in individuals with dyslexia^33^, which is in line with the reported correlation of left-handedness and dyslexia^34^. Furthermore, *RFX3* is involved in ciliogenesis which is suggested to play an important role in the aetiology of dyslexia^4^,^35^. When regarding eQTL findings for both associated SNPs rs2038137-*KIAA0319* and rs6935076-*KIAA0319*, the strongest effect on gene expression was found on *KIAA0319* itself. Effect sizes on *KIAA0319* were consistent with association level observed in our case-control analysis (Fig. 3) as the two SNPs associated in our case-control and in our meta-analysis revealed the strongest effect on *KIAA0319*. This point to a mechanism of action of these SNPs including the regulation of expression levels of *KIAA0319*. Note that the strongest SNP affecting the expression levels of *KIAA0319* is rs761100 (Supplemental Table 4). This SNP was previously associated with expressive language scores^24^ and, thus seems to be an additional reasonable candidate for future analyses.

To the best of our knowledge, SNP rs7765678-*DCDC2* was identified for the first time in a case-control setting with our study. The effect size direction was also in accordance with the association in the general population. This variant is the second variant in *DCDC2* with association in our sample as we previously found association with the intron 2 deletion of *DCDC2*^17^,^18^. Note that we did not find linkage disequilibrium with other SNPs in *DCDC2* for which association with dyslexia in a case-control cohort was previously reported, supporting novelty (Supplemental Table 1). Originally, SNP rs7765678-*DCDC2* was associated with quantitative measures of regular word reading^8^. Interestingly, for SNP rs7765678-*DCDC2* we did not found an association altered expression of *DCDC2* itself, but with levels of *MRS2* (Table 3 and Fig. 3). Consistently, the SNPs associated in our case-control cohort showed the strongest effect on expression levels of *MRS2*. *MRS2* is associated with ‘hyperphosphatasia with mental retardation syndrome’ (HPMRS)^36^ and, besides other brain regions, highly expressed in the cerebellum^26^. Future studies have to show whether this relation might be part of a pathomechamism of this SNP. Not that in general, in early periods of neuronal development expression levels need to be tightly controlled, which is relevant for dyslexia^37^.

We are aware that due to the moderate sample size our results are limited and therefore require comparison with findings from additional studies as done for the SNPs with available external data in our meta-analysis. Nevertheless, advantages of our study also exist, e.g. the homogeneity of our cohort regarding language and local recruitment. It’s also worth mentioning, that we cannot completely exclude that population stratification and cryptic relationship might have a certain effect on the analyses. However, due to our recruitment strategy focussing on the local well defined population, this bias might be considerably low.

Still, given our limitations, we suggest that for an overall conclusion about general differences in the genetic architecture of reading in the general population and clinical dyslexia, additional studies have to be done.

Since dyslexia has a polygenic background and contributions of individual genetic markers might be low, it seems promising to not only analyse the SNPs on single marker level but jointly. Encouragingly, we found that the percentage of risk alleles was higher for more SNPs than expected by chance (p-value = 0.033). Supporting, we observed a general increase of the sums of all originally reported risk alleles in cases, Fig. 2a). Importantly, this increase was not only driven by the three SNPs that associated nominally at single level as this effect persisted when analysing only non-associated SNPs (Fig. 2b). When adding SNPs analysed in previous studies to the risk score, the characteristic risk allele pattern remains (Supplemental Fig. 2). Note that given the different measures used in the original studies, we could not apply a weighted score. Our observed shift between the risk score of cases and controls appears to be very similar to shifts found when summing up highly validated risk alleles from large genome-wide studies in other complex diseases^38^,^39^. It is in line with the finding that our study had 80% power to replicate the direction of a reported risk allele with very small effect sizes down to an OR of 1.093–1.152. Hence, our power to detect a reported trend is considerably more sensitive than the power to detect a reported association (i.e., here we have 80% power to detect an OR of 1.36–1.62). To the best of our knowledge our results provide first evidence in a relevant dataset that well-known strategies to sum risk alleles across multiple genes may be also promising in the field of dyslexia. Therefore, our findings may motivate future larger collaborative studies in dyslexia targeting variants with smaller genetic effect sizes.

In summary, our work corroborates the importance of rs7765678-*DCDC2*, rs2038137-*KIAA0319*, and rs6935076-*KIAA0319* in the aetiology of dyslexia. The relevance of rs2038137-*KIAA0319*, and rs6935076-*KIAA0319* was further supported by our meta-analysis. The increased number of originally reported risk alleles in our dyslexia cases compared to controls and the replicated effect directions above chance may support a contribution to dyslexia risk for at least some of the other SNPs without associations on single marker level. Additionally, we provided functional attributes for many of these SNPs which can be included in future studies aiming to elucidate links between these genetic variants and dyslexia.

## Materials and Methods

### Ethical approval

The study was approved by the Ethics Committee of the University of Leipzig. Involvement of children in schools was approved by the Saxon Ministry of Culture and Sports. Informed and written consent was obtained from probands’ parents. All experiments were conducted in accordance with the informed consent and the approved guidelines of the Ethics Committee of the University of Leipzig.

### Sample

Our sample comprised 383 dyslexia cases and 357 controls. Dyslexia-cases were selected according to a two-stage process: In a first step, schools with special dyslexia classes were contacted. Children of these classes were tested with different psychometric tests at the end of the 2^nd^ grade. According to the admission criteria of these classes only children with a discrepancy between IQ and reading performance of at least 1.25 SD or higher get access. In a second step, students underwent additional testing to avoid the inclusion of ADHD affected probands (test d2)^40^, to ensure an IQ ≥ 85 (CFT20)^41^ and to get a quantitative measure for reading/spelling performance (KNUSPEL-L)^42^. For further cohort and test descriptions see Wilcke *et al*.^17^ and Mueller *et al*.^15^.

### SNP selection and genotyping

The literature was thoroughly screened for SNPs associated with reading or spelling in the general population. In total, six studies with 16 SNPs within five genes were identified (Table 1). The majority of these SNPs lack of replication in case-control studies. DNA was extracted from saliva or blood using standard protocols including Qiagen DNeasy Blood & Tissue Kit (Qiagen, Hilden, Germany), Macherey-Nagel NucleoMag 96 Blood Kit, the MACHEREY-NAGEL NucleoSpin^®^ 8 and 96 Blood Kit (Macherey-Nagel, Dueren, Germany), and the Oragene DNA Genotek Saliva Kit (Kanata, Ontario, Canada).

In total, 11 SNPs (rs1419228, rs9467075, rs9467076, rs1091047, rs7765678, rs6922023, rs8037376, rs8043049, rs8040756, rs1842129, rs1995402) were genotyped by mass spectrometry via iPlex (Sequenom, Hamburg, Germany). Five SNPs (rs6935076, rs2143340, rs4504469, rs2038137, rs7174102) were genotyped by mass spectrometry using the single-base extension (SBE) method GenoSNIP^43^. Primer sequences are provided in Supplemental Table 6. In detail, PCR was performed in 10 μl reaction volume according to the following conditions: Initial denaturation at 95 °C for 15 min, 45 cycles with denaturation at 95 °C for 45 s, primer hybridization at 58 °C for 45 s, elongation at 72 °C for 45 s and a final extension step at 72 °C for 5 min. The resulting product was digested with exonuclease and shrimp alkaline phosphatase to remove leftover primers and to dephosphorylate dNTPs. A SBE reaction was performed with specific primers with biotin residue and a photocleavage site^44^. The SBE reaction was performed with the purified PCR product according to the following conditions: Initial denaturation at 95 °C for 4 min, 44 cycles with denaturation at 94 °C for 10 s, primer hybridization at 60 °C for 30 s, and elongation at 72 °C for 10 s. The final SBE products were genotyped by MALDI-TOF mass spectrometry.

### Associations on single marker and haplotype level

The study was designed to detect an OR of 1.36–1.62 with a minor allele frequency (MAF) of 0.42–0.10 with β = 0.8. This power is in accordance with previously reported effect sizes^20^,^23^,^24^. Allelic associations were analysed using Chi^2^ statistics in order to detect group differences between dyslexia cases and controls. Genotypic associations were assessed by GRR estimations according to Lathrop^45^. Moreover, haplotypic effects were investigated using Haploview 4.1^46^. Associations of haplotypes were analysed using the implemented Chi^2^ approach.

To identify independent genetic effects, we pruned the selected SNPs (R^2^ = 0.5) while priority was given to SNPs with higher effect sizes (i.e. stronger allelic odds ratios) this is also known as clumping.

### Meta-analysis

We conducted a meta-analysis by integrating SNP data from dyslexia case-control and transmission/disequilibrium (TDT) studies. Studies were identified by systematically screening PubMed and Google Scholar with the keywords ‘dyslexia’ and the SNP-names.

Allele counts from cases and controls and numbers of transmitted alleles were used to estimate the individual effect sizes. ORs for TDT-studies were calculated according to Kazeem and Farrall^47^. Since between-cohort heterogeneity was expected, meta effect sizes were computed using a random effects model and effects were visualised by forest plots implemented in the metafor package^48^. Analyses were performed for all identified cohorts and in a second step stratified according to language. Newbury *et al*.^24^ analysed two different cohorts of cases with the same cohort of controls. Both analyses were included indicated with a) and b) as individual studies in the meta-analysis.

### Polygenic analyses

We computed an additive risk score composed of the sum among all individually observed risk variants, and its distribution between cases and controls was analysed. To only consider independent genetic effects identified by pruning. This avoids unintendedly increasing the weight of SNPs with other SNPs in LD. Note that this procedure is similar with the analysis of an unweighted genetic risk score using the PRSice approach^49^. This approach was performed for three different sets of SNPs: All SNPs analysed in this study (Fig. 2a), all SNPs except the ones nominally associated (Fig. 2b) and all SNPs of this study with addition of the SNPs analysed in previous studies on the same cohort (rs793862-*DCDC2*^17^*, rs807701-*DCDC2*^17^*, rs807724-*DCDC2*^17^, the intron 2 deletion of *DCDC2*^17^*, rs12533005-*FOXP2*^16^*, rs10502812-*AK131011*^15^*, rs11873029-*DYM*^15^*, rs11874896-*EPB41L3*^15^*, rs11661879-*KIAA0427*^15^*, rs1299348-*MC5R*^15^*, rs555879-*MYO5B*^15^*, rs12606138-*NEDD4L*^15^, rs8094327-*NEDD4L*^15^*, rs9461045-*KIAA0319*^18^ (SNPs marked with * show independent effects (R^2^ ≥ 0.5) and were included for analysis).

The probability of finding the observed effect directions under the NULL for the pruned SNPs was calculated via the binomial distribution. The power to detect a reported trend was calculated as previously described^50^.

In a supplementary analysis, we additionally considered for the polygenic score all dyslexia candidate SNPs previously reported in our cohort into the score, regardless whether we had found association or not. These SNPs were rs793862-*DCDC2*^17^*, rs807701-*DCDC2*^17^*, rs807724-*DCDC2*^17^, the intron 2 deletion of *DCDC2*^17^*, rs12533005-*FOXP2*^16^*, rs10502812-*AK131011*^15^*, rs11873029-*DYM*^15^*, rs11874896-*EPB41L3*^15^*, rs11661879-*KIAA0427*^15^*, rs1299348-*MC5R*^15^*, rs555879-*MYO5B*^15^*, rs12606138-*NEDD4L*^15^, rs8094327-*NEDD4L*^15^*, rs9461045-*KIAA0319*^18^. SNPs marked with * show independent effects (R^2^ ≥ 0.5) and were included for analysis. Data for all these SNPS was available for 269 cases and 357 controls.

All statistical analyses were done in R 3.2.1^51^ or perl using in-house scripts available upon request.

### Functional *in silico* characterisation

We analysed the SNPs for effects on local and distant gene expression levels (*cis* and *trans*-eQTLs) using data from published large-scale experiments^52^,^53^,^54^,^55^,^56^,^57^,^58^,^59^,^60^,^61^,^62^,^63^,^64^,^65^,^66^,^67^,^68^,^69^,^70^. These studies cover a broad range of tissues, like blood-derived cells, skin, liver and different brain regions. To identify proxy SNPs in our eQTL analysis, we used a minimum level of R^2^ ≥ 0.5, linkage data was used from 1000 Genomes reference phase 1, release V3 and from HapMap Data (Release #28, lifted over to GRCh37/hg19). We report results of the proxy SNPs that showed strongest correlation and we only considered eQTLs meeting study-wide significance level. Most analysed SNPs were located in the DYX2 locus on chromosome 6. To provide a spatial overview of these SNPs and their eQTL effects we generated a local association plot using LocusZoom^71^.

SNPs were also annotated for known and predicted local regulatory elements via ‘RegulomeDB’^25^, and tissue-specificity of gene products was analysed by ‘Human Protein Atlas’^26^.

## Additional Information

**How to cite this article**: Müller, B. *et al*. Association, characterisation and meta-analysis of SNPs linked to general reading ability in a German dyslexia case-control cohort. *Sci. Rep.* **6**, 27901; doi: 10.1038/srep27901 (2016).

## Supplementary Material

Supplementary Information

Supplementary Dataset

## Figures and Tables

**Table 1:** Reference studies for all 16 analyzed SNPs with the reported risk allele.

SNP	Chromosome	Position[bp]	General population studies			Case-control studies	
			Study	Reportedrisk allele	Effect size	Reportingassociation	Not Reportingassociation
**rs1091047-*****DCDC2***	6	24295256	Lind *et al*.^8^	G	expl. variance = 0.64%	–	–
**rs1419228-*****DCDC2***	6	24178306	Lind *et al*.^8^	G	expl. variance = 0.87%	–	–
rs9467075-*DCDC2*^*#*^	6	24205236	Lind *et al*.^8^	A	expl. variance = 0.35%	–	–
rs9467076-*DCDC2*^*#*^	6	24209255	Lind *et al*.^8^	C	expl. variance = 0.34%	–	–
**rs6922023-*****DCDC2***	6	24348117	Lind *et al*.^8^	G	expl. variance = 0.40%	–	–
**rs7765678-*****DCDC2***	6	24330544	Lind *et al*.^8^	T	expl. variance = 0.45%	–	–
**rs8037376-*****DYX1C1***	15	55768321	Paracchini *et al*.^9^	C	−0.147 SD (spelling) per risk allele	–	–
rs8043049-*DYX1C1*^*#*^	15	55777788	Paracchini *et al*.^9^	C	−0.163 SD (spelling) per risk allele	–	–
rs7174102-*DYX1C1*^*#*^	15	55719687	Paracchini *et al*.^9^	T	−0.135 SD (spelling) per risk allele	–	–
**rs8040756-*****DYX1C1***	15	55798599	Paracchini *et al*.^9^	G	−0.196 SD (reading) per risk allele	–	–
**rs2038137-*****KIAA0319***	6	24645943	Luciano *et al*.^12^; Paracchini *et al*.^13^	G	0.06 SD (reading) per copy of haplotype (rs4504469-rs2038137-rs2143340)^12^; Beta = −0.067 (adjusted reading values) per copy of haplotype (rs4504469-rs2038137-rs2143340)^13^	Harold *et al*.^19^; Cope *et al*.^20^	Couto *et al*.^21^; Scerri *et al*.^27^
rs4504469-*KIAA0319*^*#*^	6	24588884	Luciano *et al*.^12^; Paracchini *et al*.^13^	C	0.06 SD (reading) per copy of haplotype (rs4504469-rs2038137-rs2143340)^12^; Beta = −0.003 (adjusted reading values) per copy of haplotype (rs4504469-rs6935076)^13^; Beta = −0.067 (adjusted reading values) per copy of haplotype (rs4504469-rs2038137-rs2143340)^13^	Cope *et al*.^20^; Newbury *et al*.^24^; Becker *et al*.^23^	Couto *et al*.^21^; Brkanac *et al*.^22^, Harold *et al*.^19^
**rs6935076-*****KIAA0319***	6	24644322	Luciano *et al*.^12^; Paracchini *et al*.^13^	T	0.06 SD (reading) per copy of haplotype (rs4504469-rs2038137-rs2143340)^12^; Beta = −0.003 (adjusted reading values) per copy of haplotype (rs4504469-rs6935076)^13^	Cope *et al*.^20^; Couto *et al*.^21^	Harold *et al*.^19^
**rs1842129-*****NKAIN2***	6	124838090	Luciano *et al*.^11^	G	Not reported	–	–
**rs1995402-*****ROBO1***	3	79790407	Bates *et al*.^10^	A	Not reported	–	–
**rs2143340-*****TDP2***	6	24659071	Luciano *et al*.^12^ ; Paracchini *et al*.^13^	C	Expl. variance:1% (reading)^12^ ;Beta = −0.074 (adjusted reading values) per risk allele^13^; Beta = −0.067 (adjusted reading values) per copy of haplotype (rs4504469-rs2038137-rs2143340)^13^	Newbury *et al*.^24^	Cope *et al*.^20^; Couto *et al*.^21^, Scerri *et al*.^27^, Becker *et al*.^23^

Note that rs2038137 and rs4504469 were reported to associate as 3-marker-haplotype rs4504469-rs2038137-rs2143340 and rs4504469 as 2-marker-haplotype rs4504469-rs6935076 in a case-control study. SNP positions are based on HG19. Explained variances for SNPs identified by Lind *et al*.^8^ were calculated for a principal factor score computed from six reading and spelling related measures. For the other SNPs, phenotypes are given in brackets. Shown is the absolute value of the effect size as presented in the paper. SNPs with independent effects identified by pruning (R^2^ = 0.5) are bold, and SNPs without independent effects are marked with # and were tagged by the respective independent SNP shown above.

**Table 2:** Association statistics.

SNP	Reported risk allele and accordance	Major homozygous genotype		Minor homozygous genotype		Allelic association	
		p	GRR	p	GRR	p	OR
**rs1091047-*****DCDC2***	Major (acc.)	0.062	1.34 (1.0–1.8)	0.720	0.88 (0.5–1.7)	0.085	0.78 (0.6–1.0)
**rs1419228-*****DCDC2***	Minor (acc.)	0.491	0.90 (0.7–1.2)	0.051	1.77 (1.0–3.1)	0.292	1.15 (0.9–1.5)
rs9467075-*DCDC2*^*#*^	Major	0.892	1.02 (0.7–1.4)	0.481	1.29 (0.6–2.6)	0.990	1.00 (0.7–1.3)
rs9467076-*DCDC2*^*#*^	Minor (acc.)	0.985	0.99 (0.7–1.4)	0.130	1.79 (0.8–3.8)	0.767	1.05 (0.8–1.4)
**rs6922023-*****DCDC2***	Major (acc.)	0.268	1.19 (0.9–1.6)	0.186	0.61 (0.3–1.3)	0.186	0.83 (0.6–1.1)
**rs7765678-*****DCDC2***	Major (acc.)	0.035*	1.52 (1.0–2.2)	0.178	0.37 (0.1–1.6)	0.023*	0.65 (0.5–0.9)
**rs8037376-*****DYX1C1***	Minor (acc.)	0.951	1.01 (0.8–1.3)	0.314	1.21 (0.8–1.7)	0.741	1.04 (0.8–1.3)
rs8043049-*DYX1C1*^*#*^	Major	0.576	1.08 (0.8–1.4)	0.494	1.13 (0.8–1.6)	0.970	0.99 (0.8–1.2)
rs7174102-*DYX1C1*^*#*^	Minor	0.594	1.08 (0.8–1.4)	0.439	1.15 (0.8–1.6)	0.989	1.00 (0.8–1.2)
**rs8040756-*****DYX1C1***	Major (acc.)	0.305	1.19 (0.9–1.7)	0.701	1.17 (0.5–2.6)	0.38	0.87 (0.6–1.2)
**rs2038137-*****KIAA0319***	Major (acc.)	0.036*	1.35 (1.0–1.8)	0.887	0.97 (0.7–1.4)	0.132	0.85 (0.7–1.0)
rs4504469-*KIAA0319*^*#*^	Major (acc.)	0.987	0.99 (0.8–1.3)	0.562	0.90 (0.6–1.3)	0.802	0.97 (0.8–1.2)
**rs6935076-*****KIAA0319***	Minor (acc.)	0.088	0.78 (0.6–1.0)	0.036*	1.46 (1.0–2.1)	0.039*	1.25 (1.0–1.5)
**rs1842129-*****NKAIN2***	Major	0.100	0.78 (0.6–1.0)	0.845	1.04 (0.7–1.5)	0.261	1.13 (0.9–1.4)
**rs1995402-*****ROBO1***	Minor (acc.)	0.680	0.94 (0.7–1.3)	0.708	0.94 (0.7–1.3)	0.977	1.01 (0.8–1.2)
**rs2143340-*****TDP2***	Minor	0.442	1.14 (0.8–1.6)	0.665	0.84 (0.4–1.8)	0.406	0.88 (0.7–1.2)

Shown are the respective p-values and genetic relative risks (GRR) for the homozygous major allele genotype and the homozygous minor allele genotype. Allelic associations relate to the effect of the minor allele reported in Supplemental Table 7. Shown is also the accordance (acc.) of the reported risk allele from literature with the observed risk allele of our study. SNPs with independent effects identified by priority pruning (R^2^ = 0.5) are bold, and SNPs without independent effects are marked with # and were tagged by the respective independent SNP shown above.

**Table 3:** EQTL and regulatory annotations for all analysed SNPs.

SNP	eQTLs		Regulome DB
	eQTN (R^2^)	Affected gene	Predicted elements
rs1091047-*DCDC2*	rs17302582 (R^2^ = 0.79)	*ALDH5A1* (p = 0.0013)^67^	no data
rs1419228-*DCDC2*	SNP = eQTN	*MRS2* (p = 9.9E-06)^67^	Motifs-PWM (FOXC1)
rs9467075-*DCDC2*	SNP = eQTN	*MRS2* (p = 0.0027)^67^	Chromatin structure (DNase-seq, FAIRE) Protein binding-ChIPseq (RAD21, CTCF)
rs9467076-*DCDC2*	rs9460977 (R^2^ = 1.00)	*MRS2* (p = 0.0003)^67^	no data
rs6922023-*DCDC2*	SNP = eQTN	*DCDC2* (p = 3.8E-06)^69^	no data
rs7765678-*DCDC2*	SNP = eQTN	*MRS2* (p = 1.8E-07)^67^	Chromatin structure (DNase-seq, FAIRE) Protein binding-ChIPseq (HNF4A, EP300, FOXA1, TCF4, FOXA2)
rs8037376-*DYX1C1*	SNP = eQTN	*PIGB* (p = 1.9E-44)^67^*CCPG1* (p = 5.2E-20)^65^*DYX1C1* (p = 1.1E-05)^69^*DYX1C1-CCPG1* (p = 1.4E-05)^69^*RP11-139H15.1* (p = 1.7E-05)^69^*RSL24D1* (p = 0.0005)^67^	Chromatin structure (DNase-seq, FAIRE) Protein binding-ChIPseq (MEF2A)
rs8043049-*DYX1C1*	SNP = eQTN	*PIGB* (p = 2.7E-47)^67^*CCPG1* (p = 3.8E-19)^67^*DYX1C1-CCPG1* (p = 2E-05)^69^*RSL24D1* (p = 0.0025)^67^	Chromatin structure (DNase-seq, FAIRE)
rs7174102-*DYX1C1*	SNP = eQTN	*PIGB* (p = 5E-23)^67^*CCPG1* (p = 1.2E-17)^65^*DYX1C1* (p = 3.1E-06)^69^	Motifs-PWM (Plagl1) Chromatin structure (DNase-seq, FAIRE)
rs8040756-*DYX1C1*	SNP = eQTN	*CCPG1* (p = 0.00018)^55^*PIGB* (p = 1.6E-05)^67^*C15orf65* (p = 7.4E-08)	no data
rs2038137-*KIAA0319*	SNP = eQTN	*KIAA0319* (p = 1.3E-14)^69^*RP1-30M3.5* (p = 3.6E-10)^69^*ACOT13* (p = 9.9E-09)^69^*KRT8P43* (p = 3.2E-06)^69^*TDP2* (p = 8.5E-06)^69^	Motifs-Footprinting(Staf) Motifs-PWM (Staf) Chromatin structure (DNase-seq, FAIRE) Protein binding-ChIPseq (RFX3, POLR2A, E2F6)
rs4504469-*KIAA0319*	SNP = eQTN	*KIAA0319* (p = 1.3E-13)^65^*ACOT13* (p = 0.0003)^67^	no data
rs6935076-*KIAA0319*	SNP = eQTN	*KIAA0319* (p = 6.4E-14)^69^*ACOT13* (p = 7.6E-07)^69^	Chromatin structure (DNase-seq, FAIRE)
rs1842129-*NKAIN2*	no data	no data	Motifs-PWM (Lhx5, Lhx3, Lhx9)
rs1995402-*ROBO1*	no data	no data	Motifs-PWM (Bbx)
rs2143340-*TDP2*	SNP = eQTN	*ACOT13* (p = 2.6E-25)^67^*C6ORF62* (p = 2.5E-16)^67^RP1-30M3.5 (p = 2E-09)^69^	Motifs-PWM (CSBP, ISGF-3, IRF-1)

eQTL annotations include the gene whose expression levels were linked to the SNP and the study from which the evidence originates. The analyses were done for eQTLs identified in all tissues and for eQTLs identified in brain related tissue. Reported are the best-linked eQTNs (R^2^** ≥ **0.5), the linkage to the original SNP (R^2^), the affected gene and the strength of association (if an eQTL-association was reported in different studies, the strongest p-value is reported). EQTL-effects were identified in *cis* unless otherwise stated. Functional annotations classified as “predicted” and “known” from ‘RegulomeDB’ are also shown.
