# Supplementary material for: Association, characterisation and meta-analysis of SNPs linked to general reading ability in a German dyslexia case-control cohort
Source: Sci Rep. 2016 Jun 17;6:27901. doi: 10.1038/srep27901 (PMC4911550; doi:10.1038/srep27901)
Supplement: Supplementary Information [file srep27901-s1.pdf]

Association, characterisation and meta-analysis of SNPs linked to general reading ability in a German dyslexia case-control cohort

Bent Müller, Arndt Wilcke, Ivonne Czepezauer, Peter Ahnert, LEGASCREEN consortium,
Johannes Boltze, Holger Kirsten

Supplementary Informations

Supplemental Table 1. LD between analysed SNPs and SNPs known to associate with dyslexia related phenotypes including case-control studies. Test SNPs included in the study are marked with *. Shown are the tested SNPs with the respective reference, the distance to the original SNP, the linkage to the original SNP (R^2) and the position (HG19). LD is based on 1000 Genomes Pilot 1 (SNAP Proxy Search).

Original SNP	Test SNP	Distance [bp]	R^2	Chromosome	Position [bp]
rs9467075	rs9467076*[1]	4019	0.582	6	24209255
rs9467076	rs9467075*[1]	4019	0.582	6	24205236
rs7174102	rs8037376*[2]	48634	0.652	15	55768321
rs7174102	rs11629841[3]	57951	0.583	15	55777638
rs7174102	rs8043049*[2]	58101	0.583	15	55777788
rs8037376	rs11629841[3]	9317	0.904	15	55777638
rs8037376	rs8043049*[2]	9467	0.904	15	55777788
rs8037376	rs7174102*[2]	48634	0.652	15	55719687
rs8037376	rs692691[3]	7746	0.555	15	55760575
rs8043049	rs11629841[3]	150	1.000	15	55777638
rs8043049	rs8037376*[2]	9467	0.904	15	55768321
rs8043049	rs7174102*[2]	58101	0.583	15	55719687
rs2038137	rs2179515[4]	17740	1.000	6	24628203
rs2038137	rs761100[4–7]	13301	0.735	6	24632642
rs2038137	rs4504469*[4,5,8–11]	57059	0.732	6	24588884
rs4504469	rs2179515[4]	39319	0.732	6	24628203
rs4504469	rs2038137*[2,9–11]	57059	0.732	6	24645943
rs2143340	rs9461045[5,12]	10010	0.718	6	24649061
rs2143340	rs3212236[6,13]	10616	0.718	6	24648455

Supplemental Table 2. Dyslexia candidate SNPs on chromosome 3, 6 and 15. All SNPs were associated with dyslexia or a related phenotype in at least one study.

SNP	Gene & Reference	SNP	Gene & Reference
rs17819126	*DYX1C1*[2,14]	rs6802848	*ROBO1*[15]
rs3743204	*DYX1C1*[3,4,14,16]	rs685935	*DYX1C1*[2,14]
rs1842129	*NKAIN2*[17]	rs692691	*DYX1C1*[3]
rs4504469	*KIAA0319*[4,5,8–11]	rs6935076	*KIAA0319*[4,5,8,10,11,13,18]
rs7174102	*DYX1C1*[2]	rs730154	*CYP19A1*[19]
rs4130991	*ROBO1*[15]	rs7622444	*ROBO1*[15]
rs11629841	*DYX1C1*[3]	rs793862	*DCDC2*[4,7,13,20–22]
rs1387665	*ROBO1*[15]	rs8034835	*CYP19A1*[19]
rs4680960	*ROBO1*[15]	rs8040756	*DYX1C1*[2]
rs6548628	*ROBO1*[15]	rs934634	*CYP19A1*[19]
rs7614913	*ROBO1*[15]	rs9356928	*NRSN1*[18]
rs8037376	*DYX1C1*[2]	rs9461045	*TDP2* (1.1kb)[5,12]
rs8043049	*DYX1C1*[2]	rs4264688	*ROBO1*[15]
rs9467076	*DCDC2*[1]	rs7765678	*DCDC2*[1]
rs7623728	*ROBO1*[15]	rs807724	*DCDC2*[20]
rs6922023	*DCDC2*[1]	rs3178	*NRSN1*[18]
rs9467075	*DCDC2*[1]	rs1087266	*DCDC2*[20]
rs12495133	*ROBO1*[23]	rs2179515	*KIAA0319*[4,8]
rs10046	*CYP19A1*[19]	rs793842	*DCDC2*[24]
rs12636438	*ZNF385D*[25]	rs1419228	*DCDC2*[1,2]
rs6548651	*ROBO1*[15]	rs600753	*DYX1C1*[16]
rs1365152	*CLSTN2*[26]	rs807701	*DCDC2*[4,7,21,22]
rs2114167	*CLSTN2*[26]	rs2038137	*KIAA0319*[2,9–11]
rs3181238	*TDP2*[18]	rs1995402	*ROBO1*[15]
rs3212236	*TDP2 (1.7kb)*[5,6]	rs2143340	*KIAA0319*[2,5,9–11]
rs331142	*ROBO1*[23]	rs1091047	*DCDC2*[1]
rs3743205	*DYX1C1*[3–5,16,27,28]	rs761100	*KIAA0319*[4–7]

Supplemental Table 3. Allele-frequencies of the published rs4504469-rs2038137-rs2143340 haplotype and three gene-wide haplotypes including *DCDC2*, *KIAA0319* and *DYX1C1*. Frequencies are given for all probands, cases and controls.

Haplotype	Frequency (all)	Frequency (cases)	Frequency (controls)
**rs4504469-rs2038137-rs2143340**			
CGA	0.427	0.441	0.412
TTA	0.311	0.298	0.325
CGG	0.130	0.122	0.137
TGA	0.062	0.074	0.050
CTA	0.054	0.049	0.059
TGG	0.014	0.014	0.014
**rs1419228-rs9467075-rs9467076-rs7765678-rs6922023 (*DCDC2*)**			
AGTGTG	0.578	0.586	0.569
GACGTG	0.111	0.116	0.106
AGTCCA	0.071	0.053	0.09
AGTGTA	0.068	0.072	0.063
AGTCTG	0.061	0.063	0.059
GGTGTG	0.039	0.042	0.036
AGTCTA	0.022	0.021	0.022
GATGTG	0.012	0.013	0.011
AATGTG	0.011	0.007	0.015
**rs4504469-rs6935076-rs2038137 (*KIAA0319*)**			
CAG	0.328	0.346	0.309
TGT	0.313	0.298	0.328
CGG	0.229	0.219	0.241
TAG	0.058	0.067	0.048
CGT	0.05	0.046	0.055
TGG	0.018	0.02	0.015
**rs7174102-rs8037376-rs8043049-rs8040756 (*DYX1C1*)**			
ATTG	0.468	0.48	0.456
TCCG	0.286	0.294	0.278
ATTA	0.102	0.097	0.108
ACCG	0.052	0.052	0.052
TTTG	0.047	0.043	0.051
TTCG	0.012	0.01	0.015
TCCA	0.01	0.009	0.013
ATCG	0.01	0.006	0.015

Supplemental Table 4. Details of eQTL annotations. Shown are the eQTNs and their linkage (R^2) to the original SNP, the affected gene, the analysed tissue and the reference. For comparability, we report the strongest eQTN identified for each regulated gene and the respective significance level and the linkage to the original SNP and the reported eQTN. The analysis was performed for eQTLs from all tissues (left) and for eQTLs identified in brain-related tissue (right).

(See separate excel file)

Supplemental Table 5. Expression characteristics of the analyzed genes. Displayed are the protein detection levels stratified for neuronal tissue and cell type and on RNA level for the cerebral cortex.

Region	*DCDC2*	*DYX1C1*	*KIAA0319*	*NKAIN2*	*ROBO1*	*TDP2*
Protein level						
cerebellum / cells in granular layer	not detected	not detected	medium	not detected	medium	no data
cerebellum / cells in molecular layer	not detected	medium	medium	not detected	medium	no data
cerebellum / purkinje cells	not detected	medium	medium	medium	medium	no data
cerebral cortex / endothelial cells	not detected	low	low	medium	low	no data
cerebral cortex / glial cells	not detected	low	not detected	medium	high	no data
cerebral cortex / neuronal cells	low	medium	high	low	medium	no data
cerebral cortex / neuropil	not detected	not detected	medium	not detected	high	no data
hippocampus / glial cells	not detected	not detected	low	not detected	medium	no data
hippocampus / neuronal cells	not detected	medium	high	not detected	medium	no data
lateral ventricle / glial cells	not detected	medium	not detected	low	medium	no data
lateral ventricle / neuronal cells	not detected	medium	high	low	medium	no data
RNA level						
cerebral cortex	low	low	low	medium	medium	medium

Supplemental Table 6. GenoSNIP primer. Sequences for PCR primers are displayed from 5' to 3' end. The sequence of the single base extension (SBE) primer contains a biotin residue (bio) and a photocleavage linker (L).

	PCR primer forward	PCR primer reverse	SBE Primer
rs6935076	ACGTTGGATGACAGACACACCCACTCACT	ACGTTGGATGGCCTCAATAACATAGTTTCA	bioCACGCAGACA(L)GAGGAGAATGA
rs2143340	ACGTTGGATGTGGAATCAGTTGTTTCTTCA	ACGTTGGATGCATGCAATTCTTGTTAACC	bioTACAGACA AA(L)TTTAAAAGAGCCCTA
rs4504469	ACGTTGGATGTCTAATGCCTTCCCATAGT	ACGTTGGATGGTAGGAGATATGGGTAGCTC	bioGTGGACTCA(L)AGGGGGCTG
rs2038137	ACGTTGGATGAATCTTAAGAAACTCACCTTCT	ACGTTGGATGCMCAGGTACGGTATCTACTTC	bioTATTTCTCGG(L)CAGGCGC
rs7174102	ACGTTGGATGAATTAAAGATTGGAGCTGTT	ACGTTGGATGATAAACTGACTCATCTAATCTTG	bioAATCTTGT(L)GCCCCCCCA

Supplemental Table 7. Description of the analyzed SNPs. SNP-type with the minor allele of each SNP given in lower case, minor allele frequency (MAF) of cases and controls and Hardy-Weinberg-Equilibrium (HWE) in controls.

SNP-Gene	SNP-type	MAF (controls)	MAF (cases)	HWE (controls)
rs1091047-*DCDC2*	G/c	0.185	0.151	0.79
rs1419228-*DCDC2*	A/g	0.168	0.189	0.47
rs6922023-*DCDC2*	G/a	0.190	0.164	0.09
rs7765678-*DCDC2*	T/c	0.104	0.070	0.64
rs9467075-*DCDC2*	G/a	0.147	0.147	0.17
rs9467076-*DCDC2*	T/c	0.119	0.124	0.33
rs7174102-*DYX1C1*	A/t	0.370	0.369	0.88
rs8037376-*DYX1C1*	T/c	0.346	0.354	0.27
rs8040756-*DYX1C1*	G/a	0.130	0.115	0.17
rs8043049-*DYX1C1*	T/c	0.373	0.370	0.43
rs2038137-*KIAA0319*	G/t	0.386	0.348	0.49
rs4504469-*KIAA0319*	C/t	0.393	0.387	0.87
rs6935076-*KIAA0319*	G/a	0.360	0.414	0.72
rs1842129-*NKAIN2*	G/a	0.406	0.435	0.86
rs1995402-*ROBO1*	C/a	0.417	0.419	0.37
rs2143340-*TDP2*	A/g	0.154	0.139	0.32

Supplemental Figure 1. Forest plot of English studies (a) and German studies (b).

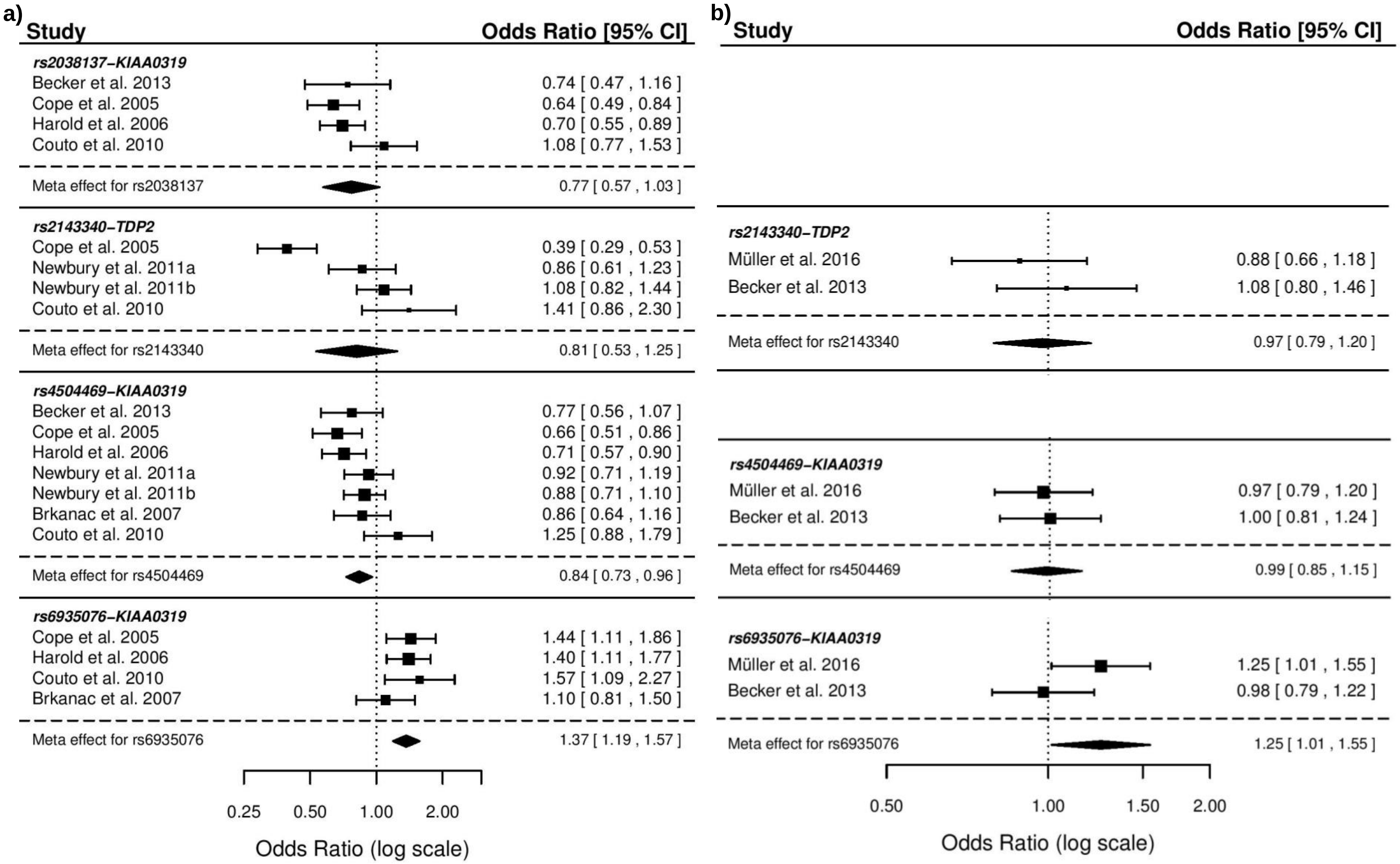

Supplemental Figure 2. Distribution of risk alleles among dyslexia-cases and controls. a) Distribution of the proportion of the sum of risk alleles from 22 SNPs comprehending all 11 (independent) SNPs associated with reading and spelling in the general population from this study and additional 11 (independent) dyslexia candidate SNPs from previously published studies of this cohort (rs793862-*DCDC2*[22], rs807701-*DCDC2*[22], the intron 2 deletion of *DCDC2*[22], rs12533005-*FOXP2*[29], rs10502812-*AK131011*[30], rs11873029-*DYM*[30], rs11874896-*EPB41L3*[30], rs11661879-*KIAA0427*[30], rs1299348-*MC5R*[30], rs555879-*MYO5B*[30], rs8094327-*NEDD4L*[30]). b) Distribution of the proportion of the sum of risk alleles including only all 11 (independent) SNPs associated with reading and spelling in the general population from this study for comparison as also shown in Figure 2a.

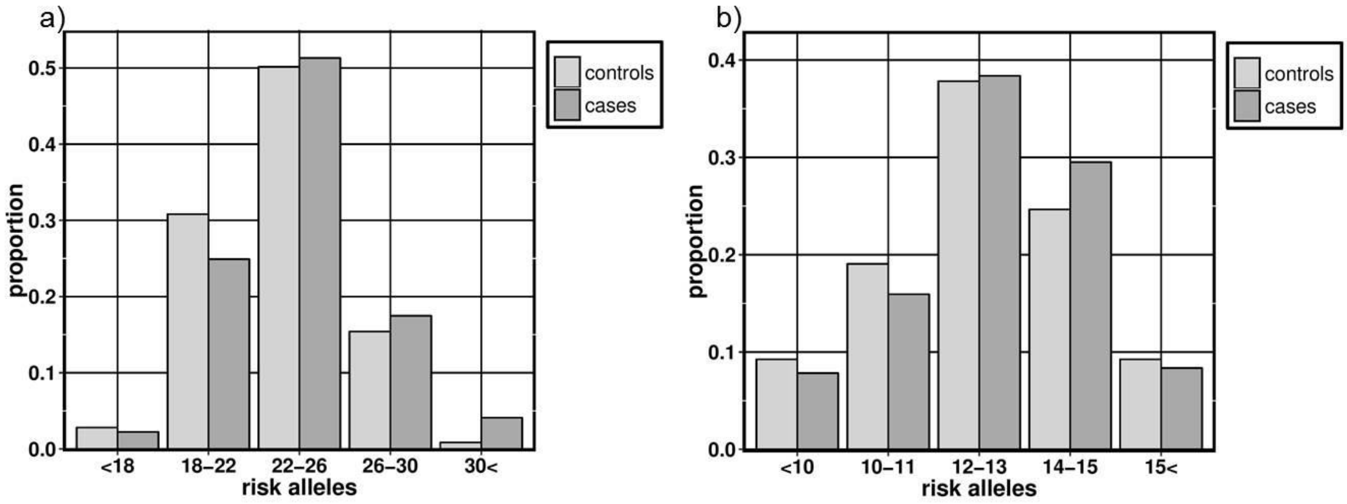

Supplemental References

1. Lind, P. a *et al.* Dyslexia and DCDC2: normal variation in reading and spelling is associated with DCDC2 polymorphisms in an Australian population sample. *Eur. J. Hum. Genet.* **18,** 668–73 (2010).
2. Paracchini, S. *et al.* Analysis of dyslexia candidate genes in the Raine cohort representing the general Australian population. *Genes. Brain. Behav.* **10,** 158–65 (2011).
3. Wigg, K. G. *et al.* Support for EKN1 as the susceptibility locus for dyslexia on 15q21. *Mol. Psychiatry* **9,** 1111–1121 (2004).
4. Becker, J. *et al.* Genetic analysis of dyslexia candidate genes in the European cross-linguistic NeuroDys cohort. *Eur. J. Hum. Genet.* 1–6 (2013). doi:10.1038/ejhg.2013.199
5. Newbury, D. F. *et al.* Investigation of dyslexia and SLI risk variants in reading- and language-impaired subjects. *Behav. Genet.* **41,** 90–104 (2011).
6. Harold, D. *et al.* Further evidence that the KIAA0319 gene confers susceptibility to developmental dyslexia. *Mol. Psychiatry* **11,** 1085–91, 1061 (2006).
7. Ludwig, K. U. *et al.* Investigation of interaction between DCDC2 and KIAA0319 in a large German dyslexia sample. *J. Neural Transm.* **115,** 1587–9 (2008).
8. Cope, N. *et al.* Strong evidence that KIAA0319 on chromosome 6p is a susceptibility gene for developmental dyslexia. *Am. J. Hum. Genet.* **76,** 581–91 (2005).
9. Francks, C. *et al.* A 77-kilobase region of chromosome 6p22.2 is associated with dyslexia in families from the United Kingdom and from the United States. *Am. J. Hum. Genet.* **75,** 1046–58 (2004).
10. Luciano, M. *et al.* A haplotype spanning KIAA0319 and TTRAP is associated with normal variation in reading and spelling ability. *Biol. Psychiatry* **62,** 811–7 (2007).
11. Paracchini, S. *et al.* Association of the KIAA0319 dyslexia susceptibility gene with reading skills in the general population. *Am. J. Psychiatry* **165,** 1576–84 (2008).
12. Dennis, M. Y. *et al.* A common variant associated with dyslexia reduces expression of the KIAA0319 gene. *PLoS Genet.* **5,** e1000436 (2009).
13. Scerri, T. S. *et al.* DCDC2, KIAA0319 and CMIP are associated with reading-related traits. *Biol. Psychiatry* **70,** 237–245 (2011).
14. Bates, T. C. *et al.* Dyslexia and DYX1C1: deficits in reading and spelling associated with a missense mutation. *Mol. Psychiatry* **15,** 1190–1196 (2010).
15. Bates, T. C. *et al.* Genetic variance in a component of the language acquisition device: ROBO1 polymorphisms associated with phonological buffer deficits. *Behav. Genet.* **41,** 50–7 (2011).
16. Dahdouh, F. *et al.* Further evidence for DYX1C1 as a susceptibility factor for dyslexia. *Psychiatr. Genet.* **19,** 59–63 (2009).
17. Luciano, M., Montgomery, G. W., Martin, N. G., Wright, M. J. & Bates, T. C. SNP sets and reading ability: testing confirmation of a 10-SNP set in a population sample. *Twin Res. Hum. Genet.* **14,** 228–32 (2011).
18. Couto, J. M. *et al.* Association of reading disabilities with regions marked by acetylated H3 histones in KIAA0319. *Am. J. Med. Genet. B. Neuropsychiatr. Genet.* **153B,** 447–62 (2010).
19. Anthoni, H. *et al.* The aromatase gene CYP19A1: several genetic and functional lines of evidence supporting a role in reading, speech and language. *Behav. Genet.* **42,** 509–27 (2012).
20. Meng, H. *et al.* DCDC2 is associated with reading disability and modulates neuronal development in the brain. *Proc. Natl. Acad. Sci. U. S. A.* **102,** 17053–8 (2005).
21. Schumacher, J. *et al.* Strong genetic evidence of DCDC2 as a susceptibility gene for dyslexia. *Am. J. Hum. Genet.* **78,** 52–62 (2006).
22. Wilcke, A. *et al.* The role of gene DCDC2 in German dyslexics. *Ann. Dyslexia* **59,** 1–11 (2009).
23. Tran, C. *et al.* Association of the ROBO1 gene with reading disabilities in a family-based analysis. *Genes. Brain. Behav.* **13,** 430–8 (2014).

24. Darki, F., Peyrard-Janvid, M., Matsson, H., Kere, J. & Klingberg, T. DCDC2 Polymorphism Is Associated with Left Temporoparietal Gray and White Matter Structures during Development. *J. Neurosci.* **34,** 14455–62 (2014).

25. Eicher, J. D. *et al.* Genome-wide association study of shared components of reading disability and language impairment. *Genes. Brain. Behav.* **12,** 792–801 (2013).

26. Roeske, D. *et al.* First genome-wide association scan on neurophysiological endophenotypes points to trans-regulation effects on SLC2A3 in dyslexic children. *Mol. Psychiatry* **16,** 97–107 (2011).

27. Taipale, M. *et al.* A candidate gene for developmental dyslexia encodes a nuclear tetratricopeptide repeat domain protein dynamically regulated in brain. *Proc. Natl. Acad. Sci. U. S. A.* **100,** 11553–8 (2003).

28. Lim, C. K. P., Ho, C. S. H., Chou, C. H. N. & Waye, M. M. Y. Association of the rs3743205 variant of DYX1C1 with dyslexia in Chinese children. *Behav. Brain Funct.* **7,** 16 (2011).

29. Wilcke, A. *et al.* Imaging genetics of FOXP2 in dyslexia. *Eur. J. Hum. Genet.* **20,** 224–9 (2012).

30. Mueller, B. *et al.* Genetic risk variants for dyslexia on chromosome 18 in a German cohort. *Genes. Brain. Behav.* 1–7 (2013). doi:10.1111/gbb.12118
